# Truncated Pneumolysin from *Streptococcus pneumoniae* as a TLR4-Antagonizing New Drug for Chronic Inflammatory Conditions

**DOI:** 10.3390/cells9051183

**Published:** 2020-05-09

**Authors:** Shun-Fu Chang, Cheng-Nan Chen, Jung-Chung Lin, Hsin-Ell Wang, Shigetarou Mori, Jia-Je Li, Chia-Kuang Yen, Ching-Yun Hsu, Chang-Phone Fung, Pele Choi-Sing Chong, Chih-Hsiang Leng, Yi-Jun Ding, Feng-Yee Chang, L. Kristopher Siu

**Affiliations:** 1 Department of Medical Research and Development, Chiayi Chang Gung Memorial Hospital, Chiayi 613, Taiwan; sfchang@cgmh.org.tw; 2 Department of Biochemical Science and Technology, National Chiayi University, Chiayi 600, Taiwan; cnchen@mail.ncyu.edu.tw (C.-N.C.); Maple313@gmail.com (C.-K.Y.); 3 Division of Infectious Diseases and Tropical Medicine, Department of Internal Medicine, Tri-Service General Hospital, National Defense Medical Center, Taipei 112, Taiwan; linjungchung1@yahoo.com.tw (J.-C.L.); uvpxyz@gmail.com (J.-J.L.); joycere18@hotmail.com (C.-Y.H.); 4 Department of Biomedical Imaging and Radiological Sciences, National Yang-Ming University, Taipei 112, Taiwan; hewang@ym.edu.tw; 5 Program in Molecular Medicine, National Yang-Ming University and Academia Sinica, Taipei 112, Taiwan; 6 Biophotonics & Molecular Imaging Research Center, National Yang-Ming University, Taipei 112, Taiwan; 7 Department of Bacteriology II, National Institute of Infectious Diseases, Tokyo 162-8640, Japan; mshige@nih.go.jp; 8 KeMyth Biotech Corporation, Innovation and Incubation Center, National Chiayi University, Chiayi 600, Taiwan; 9 Section of Infectious Diseases, Department of Medicine, Taipei Veterans General Hospital, Taipei 112, Taiwan; cpfung2000@gmail.com; 10 Graduate Institute of Basic Medical Science, China Medical University, Taichung 404, Taiwan; pchong11788@yahoo.com; 11 National Institute of Infectious Diseases and Vaccinology, National Health Research Institutes, Miaoli 350, Taiwan; leoleng@nhri.org.tw (C.-H.L.); yjding@nhri.org.tw (Y.-J.D.)

**Keywords:** chronic inflammatory disease, neutrophil activation, pneumolysin, microbial protein, Toll-like receptor 4

## Abstract

Microbial proteins have recently been found to have more benefits in clinical disease treatment because of their better-developed strategy and properties than traditional medicine. In this study, we investigated the effectiveness of a truncated peptide synthesized from the C-terminal sequence of pneumolysin, i.e., C70PLY4, in *Streptococcus pneumoniae*, in treating chronic inflammatory conditions. It has been shown that C70PLY4 significantly blocks the transendothelial migration of neutrophils and attenuates the formation of atherosclerotic plaque and the secretion of soluble forms of the intercellular adhesion molecule-1 (ICAM-1), the vascular cell adhesion molecule 1 (VCAM-1), and E-selectin in high-fat-diet/streptozotocin-induced inflammatory rats. The mechanism and the docking simulation analysis further indicated that C70PLY4 might serve as a Toll-like receptor 4 (TLR4) antagonist by competing for the binding site of MD2, an indispensable protein for lipopolysaccharide (LPS)–TLR4 interaction signaling, on the TLR4 structure. Moreover, compared to the full-length PLY, C70PLY4 seems to have no cytotoxicity in human vascular endothelial cells. Our study elucidated a possible therapeutic efficacy of C70PLY4 in reducing chronic inflammatory conditions and clarified the underlying mechanism. Thus, our findings identify a new drug candidate that, by blocking TLR4 activity, could be an effective treatment for patients with chronic inflammatory diseases.

## 1. Introduction

Microbes are identified as the major sources of natural products, protein, and medical bioremediation therapy for pharmaceutical development [1]. Microbial natural products have a long history of extensive application in the drug-related discovery of antibiotics, immunosuppressants, and anticancer agents [2,3]. Two emerging fields, i.e., the application of microbial proteins (protein therapy) and enzymes (medical bioremediation therapy), have recently been found to have more potential in clinical disease treatment because of their better-developed strategy and properties than traditional medicine [4]. Pneumolysin (PLY) from *Streptococcus pneumoniae* has been identified as a Toll-like receptor (TLR) 4 ligand, which activates NFκB translocation and enhances neutrophil transmigration or infiltration into the blood vessels. The activation and infiltration of neutrophils could further cause severe cell necrosis in the inflamed area. PLY itself is a toxin involved in virulence, especially due to its hemolytic property [5,6,7]. However, previous studies from others and us have indicated that the domain 4 of PLY (PLY4) has no or extremely low hemolytic capacity [8,9]. The concept of modifying a microbial protein to achieve a pharmaceutical target may be an effective and excellent way to identify new drugs [10,11]. Thus, at the beginning of this study, we pursue the above concept to modify PLY to act as a TLR4 inhibitor and use the modified product for the treatment of chronic inflammatory responses.

TLRs are a subgroup of the membrane pattern recognition receptors (PPRs) that sense pathogen-associated molecular patterns (PAMPs) or damage-associated molecular patterns (DAMPs) to trigger the innate immune response. PAMPs are the exogenous molecules from microorganisms, such as lipopolysaccharides (LPS), and DAMPs include endogenous molecules of cells that respond to injury or inflammation, such as heat shock protein. Thus, in addition to being the first line to sense and defend against the invading pathogens, TLRs have also been found to play a role in the development of chronic inflammatory diseases [12,13,14,15]. Among all the TLRs, TLR4, the most common one, is widely investigated because of its multiple functions and more complicated mechanism. Upon infection or stimulation, TLR4 forms a complex with its specific coreceptor, i.e., myeloid differentiation factor 2 (MD2), to induce the activation of the myeloid differentiation (MyD) 88-dependent (signaling for all the TLRs with the exception of TLR3) and the MyD88-independent (signaling only for TLR3 and TLR4) pathway [16]. TLR4/MD2 signaling further contributes to the secretion of proinflammatory cytokines and chemokines and hence leads to the development of immune and inflammatory diseases. Therefore, TLR4 has become a target for drug design and development, and some such drugs for the treatment of lung inflammation, sepsis, and rheumatoid arthritis have already entered preclinical and clinical trials [12,17,18,19,20,21]. Recently, TLR4 has been associated with other chronic inflammatory diseases, such as diabetes and atherosclerosis. A positive correlation has been shown between TLR4 and blood glucose level and atheroma formation [16,22,23]. However, the new drug investigation for these diseases still need to be further explored and developed.

In this study, we describe the use of microbial protein as a source of new drugs against chronic inflammatory diseases and report that the truncated form of PLY, i.e., C70PLY4, may block TLR4 signaling by competing with the association of TLR4 and MD2 to release the pharmaceutical potential in attenuating the neutrophil transendothelial migration, atheroma formation, and soluble adhesion molecule secretion.

## 2. Materials and Methods

### 2.1. Full-Length Pneumolysin (PLY) and Fragments

**Figure 1:**
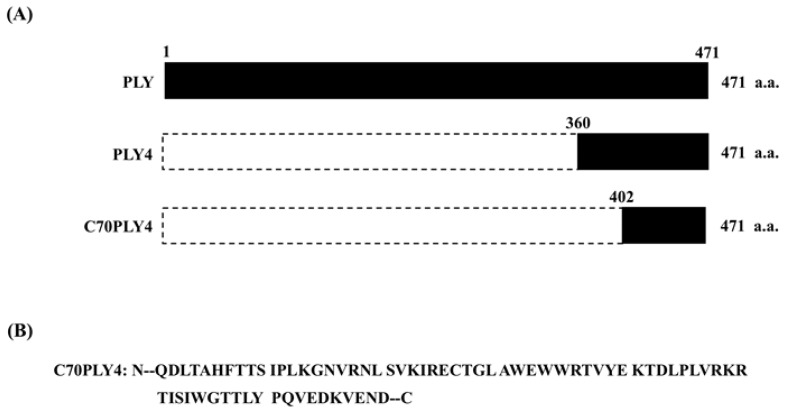
Schematic representation of the various PLY fragments. (**A**) The full-length PLY (1-471 amino acid residues) and fragments thereof, including a fragment of domain 4 of PLY (PLY4, 360-471 amino acid residues) and the C-terminal 70 amino acids of PLY4 (C70PLY4, 402-471 amino acid residues). (**B**) The amino acid sequence of C70PLY4.

The full-length pneumococcal pneumolysin (PLY, 1-471 amino acid residues) and fragments thereof, including a fragment of domain 4 (PLY4, 360-471 amino acid residues) and a C-terminal 70 amino acid fragment (C70PLY4, 402-471 amino acid residues), were produced and analyzed as shown in Figure 1.

### 2.2. Cloning, Expression, and Production of Recombinant Full-Length PLY and Domain 4 of PLY (PLY4)

The PLY4 gene was amplified using the forward primer 5’-GGAATTCCATATGAACGGAGATTTACTGCTG-3’, which contains a Nde I restriction site, and the reverse primer, 5’-CCGCTCGAGGTCATTTTCTACCTTATCTTCTAC-3’, which is complementary to the coding sequence and contains a Xho I restriction site. As a result, the C-terminal end of the recombinant protein contains an additional histidine tag, LEHHHHH. The PCR product was cloned into the expression vector pET-22b (+) (Novagen, Madison, WI, USA) using the Nde I and Xho I sites, resulting in plasmid pPLY4. The PLY4 gene was expressed in BL21 (DE3) Star from Novagen (Madison, WI, USA). The expression of recombinant PLY4 was induced with 1 mM of IPTG at 37 °C O/N, and cells were harvested by centrifugation. After the induction of PLY4, 3.6 L of cell culture was centrifuged (6400× *g* for 15 min), and the pellets were resuspended in 360 mL of phosphate-buffered saline (PBS) buffer containing 10 mM imidazole, pH 7.6. After disruption of the cells in a French Press (Constant Systems, Daventry, UK) at 27 kpsi, the cell lysates were clarified by ultracentrifugation (10,000× *g* for 60 min). The supernatant was loaded onto 9 mL of Ni-NTA resin (Qiagen, San Diego, CA, USA). The column (1.1 cm i.d. × 9.5 cm) was first washed with the homogenization buffer and then with the same buffer containing 40 mM imidazole. The PLY4 was then eluted with the homogenization buffer containing 500 mM imidazole. A polymyxin B agarose column (Pierce, Rockfold, IL, USA) was used to remove *LPS*. The amount of residual LPS in each target protein was determined with the Limulus amebocyte lysate (LAL) assay (Associates of Cape Cod, Inc., Cape Cod, MA, USA). The LPS levels were found to be below 3 EU/mg. The full-length PLY was prepared by recombinant technology in a similar manner.

### 2.3. Synthesis of Peptides

C70PLY4 was synthesized via a solid phase method using an automated peptide synthesizer (model PS-3 from Protein Technologies, Inc.), employing the fluorenylmethoxycarbonyl (Fmoc) group for α-amino group protection. The resin used was derived from a NovaSyn TGR resin with the modified Rink linker (Merck, Darmstadt, Germany). Tryptophan and lysine residues were protected with tert-butoxycarbonyl (tBoc), and arginine residues were protected with the 2,2,4,6,7-pentamethyldihydrobenzofuran-5-sulfonyl (Pbf) group. The final de-blocking step was performed with a mixture of TFA/triisopropylsilane/water (94:3:3). The crude peptides were recovered by precipitation with diethyl ether as the non-solvent and then characterized using analytical reversed-phase HPLC. Mass spectrometry analyses were performed on an Agilent 1100 Series LC/MSD high-performance ion trap mass spectrometer to ensure that the target peptide was obtained. C70PLY4 was synthesized by Almac Sciences, UK.

### 2.4. Human Umbilical Vascular Endothelial Cell (HUVEC) Culture

HUVECs were purchased from ScienCell Research Laboratories (CA, USA). The pellet was resuspended in M199 culture medium supplemented with penicillin/streptomycin and 20% fetal bovine serum (FBS), and the cells (only P3~P5) were plated onto cell culture dishes.

### 2.5. Isolation of Mouse Polymorphonuclear Neutrophils (PMNs)

The animal study was approved by the Institutional Animal Care and Use Committee of National Chiayi University (approval number: 108015). To obtain mouse neutrophils from TLR4-deficient (TLR4(-/-)) C3H/HeJ mice and wild-type C3H/HeN mice, inflammation was induced by administering 0.1 mL of uric acid solution into the peritoneal cavity of the mouse [24]. Peritoneal exudate cells were harvested 4, 18, or 24 h after injection by two lavages of the peritoneal cavity with 5 mL of cold PBS. Uric acid-induced peritoneal cells (UAPMNs) were washed by centrifugation at 200× *g* for 10 min at 4 °C. Hypotonic lysis was performed to eliminate red blood cells, and centrifugation and an additional wash were performed. The cells were then resuspended in Krebs–Ringer phosphate buffer (KRG; pH 7.3). The numbers and populations of peritoneal exudate cells were determined by staining the nuclei with Türk reagent and by cytospin centrifugation followed by May–Grünwald–Giemsa staining, respectively. The PMNs from the TLR4(-/-) C3H/HeJ mice and wild-type C3H/HeN mice were then used for the PMN transendothelial migration assay.

### 2.6. PMN Transendothelial Migration Assay

Transendothelial migration was performed using a Transwell system. Transwell filters were pre-seeded with HUVEC monolayers for 24 h and transferred to clean 24-well plates. HUVECs were washed twice with DPBS, and then 500 μL of endothelial cell medium (ECM) was added to the Transwell filter compartment, and 106 neutrophils were added to the upper chamber. Transendothelial migration was stimulated by the addition of LPS to the lower chamber at a final concentration of 1 μg/mL. Incubation was performed for 1 h at 37 °C. After the incubation, the migrated cells were collected from the lower chamber and counted in a hemocytometer in triplicate. The percentage of PMN transmigration was calculated by dividing the number of PMNs recovered from the lower chamber by the number of PMNs initially added.

### 2.7. In Vitro Protein Binding Assay for MD2 and TLR4

The recombinant TLR4 protein was diluted in PBS (2 μg/mL) and immobilized on a 96-well (100 ng/well) enzyme-linked immunosorbent assay (ELISA) plate by incubation at 37 °C for 2 h. The wells were washed three times with PBS, followed by blocking with the addition of 300 μL 5% BSA in PBS at ambient temperature overnight. Then, the plate was washed three times with PBST (PBS with 0.05% Tween 20 solution) before the addition of recombinant MD2 protein solutions at various concentrations (2-fold serial dilution from 133.3 to 0.065 nM). The ligands (MD2) and TLR4 proteins were incubated at 37 °C for 2 h, and the plate was then washed three times with PBST, followed by the addition of 100 μL of anti-MD2 antibodies diluted in 1% BSA (1:1000). After incubation at ambient temperature for 1 h, the plate was washed three times with PBST, followed by the addition of 100 μL of anti-mouse IgG-HRP antibodies diluted in 1% BSA (1:2000). After incubation at ambient temperature for 1 h, the plate was washed with PBST three times, followed by the addition of 100 μL of the HRP substrate (i.e., 3,3’,5,5’-tetramethylbenzidine, TMB) to each well. The reaction was terminated after 5 min at ambient temperature by the addition of 50 μL of the stop solution (2 N H_2_SO_4_) to each well. The optical density (OD450) in each well was determined using an ELISA plate reader. The binding affinity in nM (dissociation constant, Kd value) was computed using nonlinear regression analysis in GraphPad Prism (GraphPad Software, San Diego, CA, USA).

### 2.8. In Vitro Protein Competition Binding Assay (C70PLY4 and MD2/TLR4)

The recombinant TLR4 protein was diluted in PBS (2 μg/mL) and immobilized on a 96-well (100 ng/well) ELISA plate by incubation at 37 °C for 2 h. The wells were washed three times with PBS, followed by blocking with the addition of 300 μL of 5% BSA in PBS at ambient temperature overnight. Then, the plate was rewashed three times with PBST before the competition binding assay. The binding of MD2 (50 nM) to TLR4 was subjected to competition by the addition of various amounts (2-fold serial dilutions from 50 μM to 3.05 nM) of C70PLY4 peptide, and the residual binding was examined using anti-MD2 and anti-mouse-IgG-HRP antibodies diluted in 1% BSA, as described in the previous section (inhibitory constant, Ki value).

### 2.9. Western Blot Analysis

Cells were kept untreated as the control, treated with LPS (1 μg/mL), or co-treated with LPS (1 μg/mL) and C70PLY4 (100, 300, and 500 nM) for 24 h, and the phosphorylation of the ERK1/2 and NFκB-p65 subunits was determined by Western blot. Cells were collected by scraping and lysed with RIPA buffer containing 1% NP-40, 0.5% sodium deoxycholate, 0.1% SDS, and a protease inhibitor cocktail. The total cell lysate (50 µg of protein) was separated by SDS-polyacrylamide gel electrophoresis (PAGE) (10% running, 4% stacking) and transferred onto a polyvinylidene fluoride membrane (Immobilon P, 0.45 µm pore size). The membrane was then incubated with the designated antibodies. Immunodetection was performed using the Western-Light chemiluminescent detection system (Applied Biosystems, Foster City, CA, USA).

### 2.10. Homology Modeling and Docking Simulation

The Molecular Operating Environment (MOE; Chemical Computing Group Inc., Montreal, QC, Canada) software was used for homology modeling and docking simulation. The model of C70PLY4 was constructed by referring to the structure of the membrane-binding domain of pneumolysin from *Streptococcus pneumoniae* (PDB ID: 5CR8). The structure of human MD2 (PDB ID: 2E59) was prepared for docking simulation by the MOE Structure Preparation application (using the Amber10: EHT force-field). The binding site of MD2 was detected using the MOE Alpha Site Finder application. The docking simulation of C70PLY4 with MD2 was performed using the default parameters of the MOE Dock application. The predicted docking structure was determined considering the docking score and the structure of human MD2 in complex with lipid IVa (PDB ID: 2E59). The ribbon and protein–protein interaction diagrams were generated using PyMOL and the MOE Ligand Interactions application, respectively.

### 2.11. Inflammatory Rats of High-Fat Diet (HFD) and Low-Dose Streptozotocin (STZ) Induction

The animal study was approved by the Institutional Animal Care and Use Committee of National Chiayi University (approval number: 108015). Sprague–Dawley rats were allocated to two dietary regimens and fed either a normal diet or an HFD ad libitum for an initial period of 2 weeks. After 2 weeks of dietary manipulation, the group of rats fed an HFD were injected intraperitoneally (i.p.) with a low dose of STZ (35 mg/kg b.w.). The rats were fed their respective diets until the end of the study. The experimental animals were divided into four groups, each group comprising six rats, as follows: Group 1 served as control rats (non-inflammation control, n = 6); Group 2 served as inflammatory rats (HFD + STZ, n = 6); and Groups 3 and 4 served as inflammatory rats administered N-acetylcysteine (HFD/STZ + N-acetylcysteine, n = 6) and C70PLY4 (HFD/STZ + C70PLY4, n = 6), respectively, in aqueous suspension i.p. for 30 days. At the end of the experiment, the animals were sacrificed, and aorta and blood samples were obtained for hematoxylin and eosin staining and the ELISA assays.

### 2.12. Hematoxylin and Eosin (HE) Staining

The ascending part of the aorta was excised and kept in 10% buffered formalin for 24 h. The specimens were routinely embedded in paraffin blocks and cut into transverse sections (3 µm). The slides were stained with HE. The specimens were analyzed under a light microscope.

### 2.13. ELISA Assays

The levels of soluble forms of intercellular adhesion molecule (ICAM)-1, vascular cell adhesion molecule (VCAM)-1, and E-selectin in serum were determined using sandwich ELISA (R&D) according to the manufacturer’s protocols.

### 2.14. Cell Viability Assay

Cell viability was detected using the methanethiosulfonate (MTS) assay. In brief, 2 × 10^4^ HUVECs were seeded into 48-well plates in 100 µL of ECM (ScienCell Research Laboratory, USA) per well and incubated for 24 h. The medium was removed, and the cells were then incubated with medium containing 2% FBS in the absence or presence of various peptides, such as PLY, PLY4, or C70PLY4, at concentrations of 1, 10, 100, or 1000 nM. After 24 h of incubation, the medium was removed, and 20 µL MTS reagents (20%) were added to each well in a final volume of 100 µL and incubated for 1 h. The optical density was measured at 490 nm using a plate reader.

### 2.15. Cell Apoptosis

Cell apoptosis was detected using commercial terminal deoxynucleotidyl transferase-dUTP nick end labeling (BioVision) and caspase-3 activity kits (Biotium). In brief, after incubation with M199 medium in the presence or absence of 100 nM concentrations of C70PLY4 at 37 °C and 5% CO_2_ for 1 h, the HUVECs were washed with PBS and fixed with fixation buffer on ice for 20 min. The cells were then soaked in 70% alcohol for 1 h. After being washed twice with washing buffer, the cells were incubated in DNA labeling solution for 60 min at 37 °C and then treated with rinse buffer and incubated with antibody buffer for 30 min at room temperature. The cell nuclei were stained with propidium iodide for 30 min. Samples were observed under a fluorescence microscope and photographed with a charge-coupled device (CCD) camera. The living cells exhibited red fluorescence, while apoptotic cells showed green fluorescence. For the caspase-3 activity assay, after 100 nM concentrations of C70PLY4 treatments, the HUVECs were washed with PBS and then reacted with 1 mM Caspase-3 Enzyme Substrate for 30 min at room temperature. The cells were then observed under a fluorescence microscope and photographed with a CCD camera. The cells with caspase-3 activity exhibited green fluorescence.

### 2.16. Statistical Analysis

The results are expressed as the mean ± standard error of the mean (SEM). Statistical analysis was done using the independent Student t-test for two groups of data and analysis of variance (ANOVA) followed by Scheffe’s test for multiple comparisons. A *p*-value less than 0.05 was considered significant.

## 3. Results

### 3.1. C70PLY4 Targets TLR4 to Inhibit the LPS-Activated Transendothelial Migration of PMNs Isolated from TLR4-Wild-Type Mice

**Figure 2:**
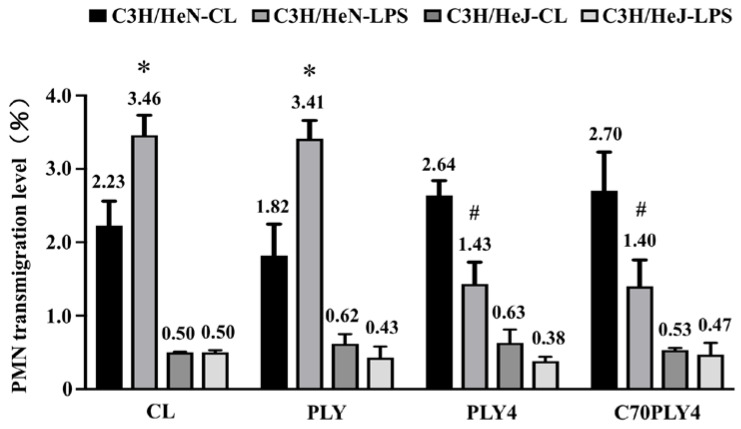
C70PLY4 targets TLR4 to inhibit the LPS-activated transendothelial migration of PMNs isolated from TLR4-wild-type mice. PMNs were isolated from C3H/HeN (TLR4-wild-type) and C3H/HeJ (TLR4-deficient) mice. The isolated PMNs were treated with PBS to serve as a control or with various PLY fragments, including PLY, PLY4, and C70PLY4, and then the isolated PMNs were added to the Transwell upper chamber cultured with HUVECs. LPS (1 μg/mL) was added to the lower chamber to attract the transendothelial migration of the PMNs. The capability of PMN transendothelial migration was determined by collecting the PMNs in the lower chamber. *, *p* < 0.05 vs. TLR4-wild-type PMNs without LPS priming. #, *p* < 0.05 vs. TLR4-wild-type PMNs treated with LPS and PLY.

We determined the effect of different PLY truncated forms (see Materials and Methods—Section 2.1, and Figure 1) on inflammation by using the PMN transmigration assay and examined whether TLR4 senses these PLY stimulations. PMNs were isolated from C3H/HeN (TLR4-wild-type) and C3H/HeJ (TLR4-deficient) mice. The isolated PMNs were treated with PBS to serve as a control or treated with various PLY fragments, i.e., PLY, PLY4, or C70PLY4, and then the isolated PMNs were added to a Transwell upper chamber cultured with HUVECs. LPS (1 μg/mL) was added to the lower chamber to attract the transendothelial migration of the PMNs. LPS-primed PMNs isolated from TLR4-wild-type mice showed a higher transendothelial migration capability than PMNs without LPS priming (Figure 2). The treatment of TLR4-wild-type PMNs with PLY4 or C70PLY4 significantly inhibited the LPS-induced transendothelial migration effect compared with that produced by treatment with PLY. In contrast, LPS did not induce the transendothelial migration of PMNs isolated from TLR4-deficient mice. Moreover, treating TLR4-deficient PMNs with PLY, PLY4, or C70PLY4 showed no further effect.

### 3.2. C70PLY4 Competes with MD2 for the Binding Site on TLR4 In Vitro and Inhibits LPS-Induced ERK1/2 and NF-κB Phosphorylation in HUVECs

**Figure 3:**
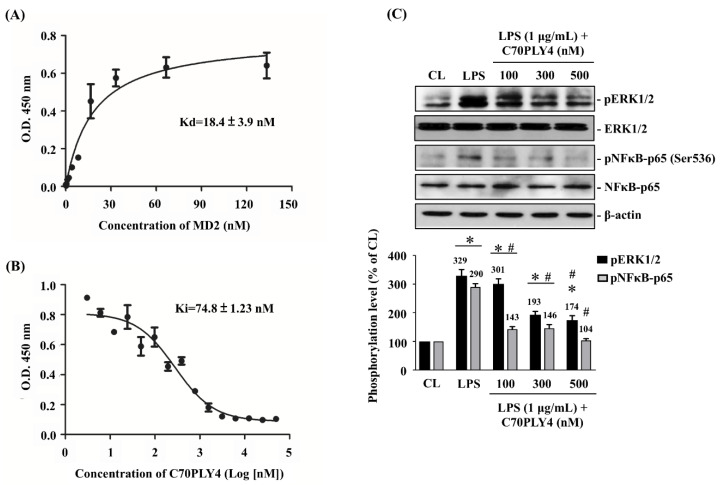
C70PLY4 competes with MD2 for the binding site on TLR4 in vitro and inhibits LPS-induced ERK1/2 and NF-κB phosphorylation in HUVECs. (**A**) TLR4 (2 μg/mL) was coated onto a 96-well plate. MD2 was added to the wells at various concentrations (2-fold serial dilutions from 133.3 to 0.065 nM), and the Kd value was determined. (**B**) TLR4 (2 μg/mL) was coated onto a 96-well plate. Mixtures of MD2 (50 nM) and various concentrations (2-fold serial dilutions from 50 μM to 3.05 nM) of C70PLY4 were added to the wells, and the Ki value was determined. (**C**) The HUVECs were kept untreated as a control, treated with LPS (1 μg/mL) alone for 24 h, or co-treated with LPS (1 μg/mL) and C70PLY4 (100, 300, and 500 nM) for 24 h, and the protein phosphorylation of the ERK1/2 and NFκB-p65 subunit were examined. The results (**C**, upper panel) are representative of three independent experiments with similar results. The data for (**A**), (**B**) and (**C**, down panel) are the mean ± SEM from at least three independent experiments. *, *p* < 0.05 vs. control cells. #, *p* < 0.05 vs. LPS-only-treated cells.

**Figure 4:**
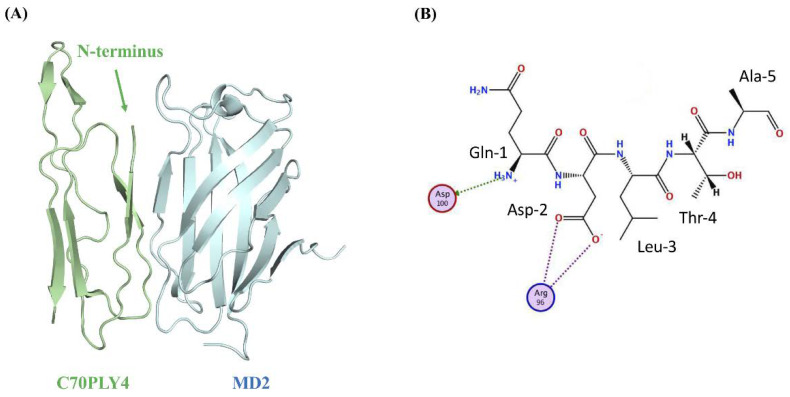
Protein docking simulation predicts the structure of C70PLY4 and MD2 interaction. (**A**) The structures of C70PLY4 and MD2 are shown in green and blue, respectively. The N-terminus of C70PLY4 is indicated. (**B**) The basic and acidic residues of MD2 are indicated by blue and red circles, respectively. The solvent and ionic interactions are indicated by green arrows and purple dotted lines, respectively. The residue numbers of C70PLY4 are indicated.

MD2, an extracellular protein that is associated with the extracellular domain of TLR4, is indispensable for the interaction of LPS and TLR4 [25]. Therefore, we examined the role of C70PLY4 in TLR4 and TLR4-elicited signaling by an in vitro MD2-TLR4 interaction competition assay and Western blot. TLR4 (2 μg/mL) was coated onto a 96-well plate, and MD2 was added to the wells at various concentrations (2-fold serial dilutions from 133.3 to 0.065 nM). The results showed that MD2 has a high binding affinity to TLR4 with a Kd (dissociation constant) value of 18.4 ± 3.9 nM (Figure 3A). In the competition assay, MD2 (50 nM) was mixed with various concentrations (2-fold serial dilutions from 50 μM to 3.05 nM) of C70PLY4 before being added to the TLR4-coated well. C70PLY4 showed a dose-dependent blocking capability in the interaction between MD2 and TLR4 with a Ki (inhibitory constant) value of 74.8 nM (Figure 3B). Moreover, MD2 could be completely out-competed by 6.25 μM of C70PLY4. We further determined whether C70PLY4 affects LPS/TLR4-induced signaling, including ERK1/2 protein and the NFκB-p65 subunit, in HUVECs. Cells were kept untreated as a control, treated with LPS (1 μg/mL) alone for 24 h, or co-treated with LPS (1 μg/mL) and C70PLY4 (100, 300, and 500 nM) for 24 h, and the protein phosphorylation of ERK1/2 and the NFκB-p65 subunit was examined. LPS induced protein phosphorylation of both ERK1/2 and the NFκB-p65 subunit in HUVECs (Figure 3C and Supplementary Figure S1). However, C70PLY4 significantly inhibited the effect of LPS on ERK1/2 and NFκB-p65 subunit phosphorylation in a dose-dependent manner (Figure 3C). Next, the Molecular Operating Environment (MOE) software was employed to predict the docking modes of C70PLY4 and MD2. The docking simulation analysis of the C70PLY4–MD2 interaction suggested that the top two residues, i.e., Gln-1 and Asp-2, at the N-terminus of C70PLY4 could play an important role in the binding of C70PLY4 to MD2 (Figure 4A,B). The binding of Gln-1 in C70PLY4 to Asp-100 in MD2 could be mediated by solvent interactions, while the binding of Asp-2 in C70PLY4 to Arg-96 in MD2 could be mediated by ionic interactions. Moreover, it was shown that Ser-53 and Trp-55 in C70PLY4 could bind to Arg-90 and Val-93 in MD2, respectively.

### 3.3. C70PLY4 Attenuates Atherosclerosis in HFD/STZ-Induced Inflammatory Rats

**Figure 5:**
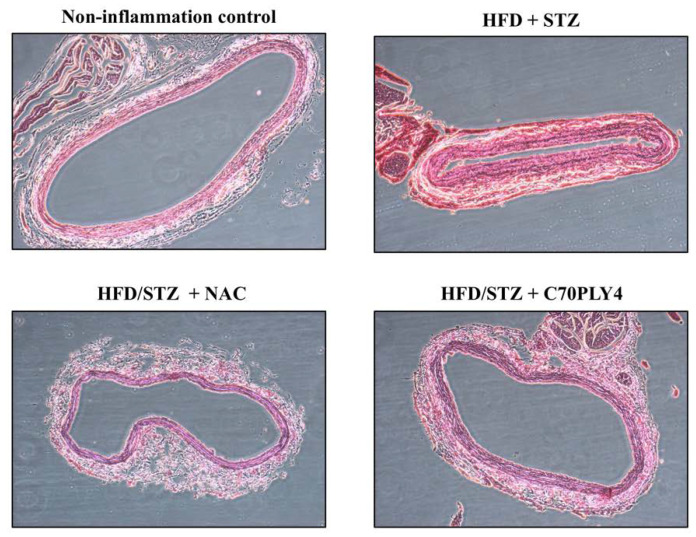
C70PLY4 attenuates atherosclerosis in streptozotocin (STZ)-injected high-fat diet (HFD)-fed rats. The rats were divided into four groups as follows: control rats (non-inflammation control), inflammatory rats (HFD + STZ), and inflammatory rats administered with NAC (HFD/STZ + NAC) or C70PLY4 (HFD/STZ + C70PLY4). The level of atherosclerotic plaque formation in the aorta was analyzed using HE staining.

Atherosclerosis is a chronic inflammatory disease. We determined whether C70PLY4 exhibits a therapeutic effect of attenuating the formation of atherosclerotic plaque in HFD/STZ-induced inflammatory rats. The rats were divided into four groups as follows: control rats (non-inflammation control); inflammatory rats (HFD + STZ); inflammatory rats administered with N-acetylcysteine (NAC), a reduced thiol that can be used to improve the atherosclerotic status (HFD/STZ + NAC); and inflammatory rats administered with C70PLY4 (HFD/STZ + C70PLY4). The level of atherosclerotic plaque formation in the aorta was analyzed using HE staining. The rats treated with HFD and STZ exhibited neointima formation in the aorta and developed atherosclerosis (Figure 5). However, the treatment of HFD/STZ-induced inflammatory rats with NAC significantly ameliorated this effect. Surprisingly, the treatment of HFD/STZ-induced inflammatory rats with C70PLY4 also significantly attenuated the neointima formation in the aorta.

### 3.4. C70PLY4 Inhibits the Secretion of Soluble ICAM-1, VCAM-1, and E-Selectin in HFD/STZ-Induced Inflammatory Rats

**Figure 6:**
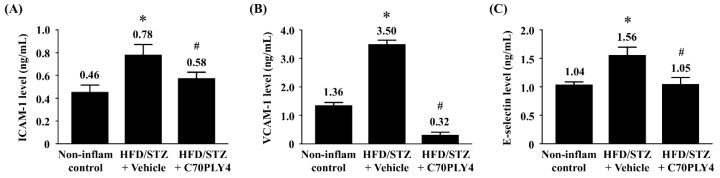
C70PLY4 inhibits the secretion of soluble ICAM-1, VCAM-1, and E-selectin in STZ-injected HFD-fed rats. The rats were divided into three groups as follows: control rats (Non-inflammatory control), inflammatory rats (HFD/STZ + vehicle), and inflammatory rats administered with C70PLY4 (HFD/STZ + C70PLY4). The secretion of (**A**) soluble ICAM-1, (**B**) soluble VCAM-1, and (**C**) soluble E-selectin in the serum was determined. Data are the mean ± SEM from three independent experiments. *, *p* < 0.05 vs. non-DM control. #, *p* < 0.05 vs. STZ-treated rats.

The soluble forms of adhesion molecules, including ICAM-1, VCAM-1, and E-selectin, induce inflammatory activity in the dysfunctional endothelium in patients with atherosclerosis and diabetes [26,27,28,29]. By using the ELISA assay, we further determined whether C70PLY4 affects the secretion of these three adhesion molecules in HFD/STZ-induced inflammatory rats. It was shown that the serum levels of soluble ICAM-1 (Figure 6A), VCAM-1 (Figure 6B), and E-selectin (Figure 6C) in HFD/STZ-induced inflammatory rats increased significantly (compared with those in the non-inflammatory control rats), whereas the treatment of HFD/STZ-induced inflammatory rats with C70PLY4 inhibited this effect.

### 3.5. C70PLY4 Does Not Affect Viability, Apoptosis, or Caspase 3 Activity in HUVECs

**Figure 7:**
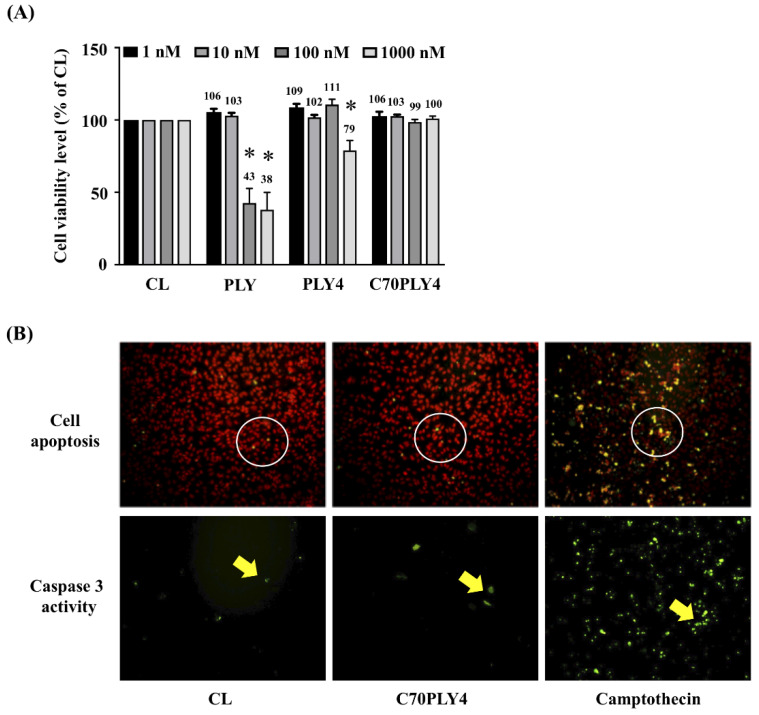
C70PLY4 does not affect the viability, apoptosis, or caspase 3 activity in human umbilical vascular endothelial cells (HUVECs). (**A**) Cells were kept untreated as a control or treated with PLY, PLY4, or C70PLY4 at a concentration of 1, 10, 100, or 1000 nM for 18 h, and the cell viability was detected by an MTS assay. (**B**) Cell apoptosis and caspase 3 activity were also detected in HUVECs under C70PLY4 treatment. Cells were kept untreated as a control or treated with C70PLY4 (100 nM) or camptothecin for 18 h, and the cell apoptosis (upper panel) and caspase 3 activity (lower panel) in the HUVECs were detected. Data in (**A**) are the mean ± SEM from three independent experiments. *, *p* < 0.05 vs. control cells.

Finally, we determined the cytotoxicity of C70PLY4 in HUVECs. Cells were kept untreated as a control or treated with various PLY fragments (see Materials and Methods—Section 2.1, and Figure 1), i.e., PLY, PLY4, or C70PLY4, at a concentration of 1, 10, 100, or 1000 nM for 24 h, and the cell viability was detected by an MTS assay. The results showed that PLY was toxic to cells when the concentration was higher than 100 nM, as less than ~40% of the cells survived; PLY4 was slightly toxic to cells when the added concentration was higher than 1000 nM; in contrast, C70PLY4 showed no cytotoxic effects on the cells even at an added concentration of 1000 nM (Figure 7A). Cell apoptosis and caspase 3 activity in HUVECs under C70PLY4 treatment were also examined. Cells were kept untreated as a control or treated with C70PLY4 (100 nM) or camptothecin, a known apoptosis inducer, for 24 h, and the resulting cell apoptosis and caspase 3 activity in the HUVECs were detected. C70PLY4 did not elicit apoptosis or caspase 3 activity in HUVECs compared with the control cells, whereas the camptothecin-treated cells exhibited approximately 60% induction of apoptosis and caspase 3 activity (Figure 7B).

## 4. Discussion

Microbes have recently been emphasized as the sources of new therapy, and more than 130 microbial proteins have already been used clinically, with many more having been used in clinical trials [1,4]. In addition to the finding of their great clinical benefits, the shorter time needed for research and development and to obtain Federal Drug Authority approval as well as the easier patent application process because of their structure and function novelty make the microbial proteins a popular source for new drug investigation in the pharmaceutical industry [4]. PLY is a well-known virulence factor that disrupts tissue barriers and then facilitates bacterial multiplication by breaching host defenses and immune responses [5,6,7]. However, it has been further found that the domain 4 of PLY (PLY4) has no or extremely low cytotoxic efficacy [8,9]. In this study, we found that the truncated form of PLY4, i.e., C70PLY4, could play a potentially therapeutic role against inflammatory conditions. Systematic experiments demonstrated the following: (i) C70PLY4 significantly inhibits PMN transendothelial migration, atherosclerotic plaque formation, and soluble ICAM-1, VCAM-I, and E-selectin secretion. (ii) These inhibitory effects of C70PLY4 might be exerted via inhibiting TLR4 activity by competing with MD2 for its binding site on TLR4. (iii) In cytotoxicity testing, C70PLY4 showed no cytotoxic effect on endothelial cells, in contrast to full-length PLY and PLY4. Our study clearly elucidated a possible therapeutic efficacy of C70PLY4 in reducing the chronic inflammatory status in different in vivo and in vitro experimental models and clarified the underlying mechanism.

Neutrophils, the most abundant type of leukocytes, have traditionally been indicated as the first line against the invaded pathogens and therefore have been involved in initiating the acute inflammation. In this case, neutrophil activation is beneficial to recovering the tissue homeostasis. However, accumulating evidence has found another crucial role of neutrophils in chronic inflammation initiated by the unlimited original stimulus and inflammation reprogram status, which eventually results in the uncovered tissue homeostasis and leads to chronic diseases, including cancer, neurodegeneration (Alzheimer’s disease), chronic obstructive pulmonary disease, atherosclerosis, arthritis, diabetes, and colitis [30,31,32,33,34]. During the development of these chronic diseases, neutrophils infiltrate into the inflamed sites to try to rescue the lesions, but they fail because of the unfailing stimulus, such as hyperlipidemia and hyperglycemia [30,35,36]. In these conditions, activated neutrophils roll and adhere to the host tissues/cells using the hosts’ adhesion molecules as well as their own, constantly secreting and/or stimulating the host cells to secrete the tissue-damaging proteases, cytokines, and chemoattractants, and consequently result in the tissue lesions [36]. Thus, it has recently been suggested that neutrophil counts are a potential predictor of chronic inflammatory diseases. The subsequent studies have also demonstrated a positive correlation between neutrophil counts and atherosclerosis progression [30,37]. In the present study, we determined and demonstrated the anti-inflammatory pharmaceutical property of C70PLY4 by using the PMN transendothelial migration assay and using the rats with HFD/STZ-induced hyperlipidemia and hyperglycemia. The results pertaining to blocking PMN migration and attenuating atheroma formation and soluble adhesion molecule secretion provide a strong possibility for the future development of C70PLY4 as a drug to treat chronic inflammatory diseases.

Recently, targeting TLR4 activity and expression in drug design and development for the prevention and treatment of diseases has attracted growing interest because of the clear understanding of TLR4 signaling in initiating chronic and low-grade inflammatory and autoimmune responses, which include obesity, infection, rheumatoid arthritis, cancer, diabetes, and cardiovascular diseases [12,13,14,15,16,22,23]. In such case, scientists have also tried to find a single protein that could achieve a dramatic therapeutic outcome for these various diseases and TLR4 has been considered one of the best candidates [12,38]. New drugs developed to inhibit TLR4 activity are already in various phases of preclinical and clinical trials for treating acute and chronic inflammation, colitis, sepsis, chronic pain, and addiction withdrawal [12,17,18,19,20,21]. In animal studies, TLR4 activity inhibition has also been demonstrated to reduce the levels of atherosclerosis and myocardial inflammation [39,40]. Moreover, supporting these results, our data have also shown that the inhibitory effect of C70PLY4 on chronic inflammatory conditions occurs through blocking the TLR4 activity via competing with MD2 for its binding site on TLR4. We therefore reasonably suggest that TLR4 might be that incredible single protein candidate. Thus, considering our results and those of others, although the evidence remains limited, a suitable drug design, such as C70PLY4, targeting TLR4 activity might provide an opportunity to effectively treat the majority of chronic inflammatory and autoimmune diseases in the future.

This study has reported a potential therapeutic application of the newfound C70PLY4 peptide, a truncated form of PLY, in anti-chronic inflammatory disease, including atherosclerosis, in HFD/STZ-induced inflammatory rat models, which might be achieved via inhibiting TLR4 activity by competing with MD2 for its binding site on TLR4. Our findings identify a new drug candidate and therefore provide a possible novel therapeutic strategy through inhibiting TLR4 activity in patients with chronic inflammatory and autoimmune diseases.

## Supplementary Materials

The following are available online at https://www.mdpi.com/2073-4409/9/5/1183/s1: Figure S1: Gel images of Western blot in Figure 3C.

## References

[1] Singh B.K., Macdonald C.A. (2010). Drug discovery from uncultivable microorganisms. Drug Discov. Today.

[2] Huang T., Lin S. (2017). Microbial Natural Products: A Promising Source for Drug Discovery. J. Appl. Microbiol. Biochem..

[3] Newman D.J., Cragg G.M. (2016). Natural products as sources of new drugs from 1981 to 2014. J. Nat. Prod..

[4] Leader B., Baca Q.J., Golan D.E. (2008). Protein therapeutics: A summary and pharmacological classification. Nat. Rev. Drug Discov..

[5] Hirst R.A., Kadioglu A., O’callaghan C., Andrew P.W. (2004). The role of pneumolysin in pneumococcal pneumonia and meningitis. Clin. Exp. Immunol..

[6] Rossjohn J., Gilbert R.J., Crane D., Morgan P.J., Mitchell T.J., Rowe A.J., Andrew P.W., Paton J.C., Tweten R.K., Parker M.W. (1998). The molecular mechanism of pneumolysin, a virulence factor from Streptococcus pneumoniae. J. Mol. Biol..

[7] Rubins J.B., Janoff E.N. (1998). Pneumolysin: A multifunctional pneumococcal virulence factor. J. Lab. Clin. Med..

[8] Baba H., Kawamura I., Kohda C., Nomura T., Ito Y., Kimoto T., Watanabe I., Ichiyama S., Mitsuyama M. (2001). Essential role of domain 4 of pneumolysin from Streptococcus pneumonia in cytolytic activity as determined by truncated proteins. Biochem. Biophys. Res. Commun..

[9] Chiu F.F., Leng C.H., Ding Y.J., Chang J.C., Chang L.S., Lien S.P., Chen H.W., Siu L.K., Liu S.J. (2019). Domain 4 of pneumolysin from Streptococcus pneumoniae is a multifunctional domain contributing TLR4 activating and hemolytic activity. Biochem. Biophys. Res. Commun..

[10] Chin Y.W., Balunas M.J., Chai H.B., Kinghorn A.D. (2006). Drug discovery from natural sources. AAPS J..

[11] Gerwick W.H., Fenner A.M. (2013). Drug discovery from marine microbes. Microb. Ecol..

[12] Hennessy E.J., Parker A.E., O’Neill L.A. (2010). Targeting Toll-like receptors: Emerging therapeutics?. Nat. Rev. Drug Discov..

[13] Kawai T., Akira S. (2009). The roles of TLRs, RLRs and NLRs in pathogen recognition. Int. Immunol..

[14] Tsan M.F., Gao B. (2004). Endogenous ligands of Toll-like receptors. J. Leukoc. Biol..

[15] Gay N.J., Symmons M.F., Gangloff M., Bryant C.E. (2014). Assembly and localization of Toll-like receptor signalling complexes. Nat. Rev. Immunol..

[16] Roshan M.H., Tambo A., Pace N.P. (2016). The Role of TLR2, TLR4, and TLR9 in the Pathogenesis of Atherosclerosis. Int. J. Inflam..

[17] Achek A., Yesudhas D., Choi S. (2016). Toll-like receptors: Promising therapeutic targets for inflammatory diseases. Arch. Pharm. Res..

[18] Bennett-Guerrero E., Grocott H.P., Levy J.H., Stierer K.A., Hogue C.W., Cheung A.T., Newman M.F., Carter A.A., Rossignol D.P., Collard C.D. (2007). A phase II, double-blind, placebo-controlled, ascending-dose study of Eritoran (E5564), a lipid A antagonist, in patients undergoing cardiac surgery with cardiopulmonary bypass. Anesthesia Analg..

[19] Wasan K.M., Risovic V., Sivak O., Lee S.D., Mason D.X., Chiklis G.R., McShane J., Lynn M., Wong N., Rossignol D.P. (2008). Influence of plasma cholesterol and triglyceride concentrations and eritoran (E5564) micelle size on its plasma pharmacokinetics and ex vivo activity following single intravenous bolus dose into healthy female rabbits. Pharm. Res..

[20] Ii M., Matsunaga N., Hazeki K., Nakamura K., Takashima K., Seya T., Hazeki O., Kitazaki T., Iizawa Y. (2006). A novel cyclohexene derivative, ethyl (6R)-6-[N-(2-Chloro-4-fluorophenyl)sulfamoyl]cyclohex-1-ene-1-carboxylate (TAK-242), selectively inhibits toll-like receptor 4-mediated cytokine production through suppression of intracellular signaling. Mol. Pharmacol..

[21] Ungaro R., Fukata M., Hsu D., Hernandez Y., Breglio K., Chen A., Xu R., Sotolongo J., Espana C., Zaias J. (2009). A novel Toll-like receptor 4 antagonist antibody ameliorates inflammation but impairs mucosal healing in murine colitis. Am. J. Physiol. Gastrointest Liver Physiol..

[22] Yin J., Peng Y., Wu J., Wang Y., Yao L. (2014). Toll-like receptor 2/4 links to free fatty acid-induced inflammation and β-cell dysfunction. J. Leukoc Biol..

[23] Dasu M.R., Devaraj S., Zhao L., Hwang D.H., Jialal I. (2008). High glucose induces Toll-like receptor expression in human monocytes: Mechanism of activation. Diabetes.

[24] Rabes A., Suttorp N., Opitz B. (2016). Inflammasomes in Pneumococcal Infection: Innate Immune Sensing and Bacterial Evasion Strategies. Curr. Top Microbiol. Immunol..

[25] Kobayashi M., Saitoh S., Tanimura N., Takahashi K., Kawasaki K., Nishijima M., Fujimoto Y., Fukase K., Akashi-Takamura S., Miyake K. (2006). Regulatory roles for MD-2 and TLR4 in ligand-induced receptor clustering. J. Immunol..

[26] Clausen P., Jacobsen P., Rossing K., Jensen J.S., Parving H.H., Feldt-Rasmussen B. (2000). Plasma concentrations of VCAM-1 and ICAM-1 are elevated in patients with Type 1 diabetes mellitus with microalbuminuria and overt nephropathy. Diabet Med..

[27] Sato J., Kinugasa M., Satomi-Kobayashi S., Hatakeyama K., Knox A.J., Asada Y., Wierman M.E., Hirata K., Rikitake Y. (2014). Family with sequence similarity 5, member C (FAM5C) increases leukocyte adhesion molecules in vascular endothelial cells: Implication in vascular inflammation. PLoS ONE.

[28] Herder C., Baumert J., Zierer A., Roden M., Meisinger C., Karakas M., Chambless L., Rathmann W., Peters A., Koenig W. (2011). Immunological and cardiometabolic risk factors in the prediction of type 2 diabetes and coronary events: MONICA/KORA Augsburg case-cohort study. PLoS ONE.

[29] Kalofoutis C., Piperi C., Kalofoutis A., Harris F., Phoenix D., Singh J. (2007). Type II diabetes mellitus and cardiovascular risk factors: Current therapeutic approaches. Exp. Clin. Cardiol..

[30] Soehnlein O., Steffens S., Hidalgo A., Weber C. (2017). Neutrophils as protagonists and targets in chronic inflammation. Nat. Rev. Immunol..

[31] Mayadas T.N., Cullere X., Lowell C.A. (2014). The multifaceted functions of neutrophils. Annu. Rev. Pathol..

[32] Coffelt S.B., Wellenstein M.D., de Visser K.E. (2016). Neutrophils in cancer: Neutral no more. Nat. Rev. Cancer.

[33] Hoenderdos K., Condliffe A. (2014). The neutrophil in chronic obstructive pulmonary disease. Am. J. Respir. Cell Mol. Biol..

[34] Wright H.L., Moots R.J., Edwards S.W. (2014). The multifactorial role of neutrophils in rheumatoid arthritis. Nat. Rev. Rheumatol..

[35] Bian Z., Guo Y., Ha B., Zen K., Liu Y. (2012). Regulation of the inflammatory response: Enhancing neutrophil infiltration under chronic inflammatory conditions. J. Immunol..

[36] Wright H.L., Moots R.J., Bucknall R.C., Edwards S.W. (2010). Neutrophil function in inflammation and inflammatory diseases. Rheumatology.

[37] Friedman G.D., Klatsky A.L., Siegelaub A.B. (1974). The leukocyte count as a predictor of myocardial infarction. N. Engl. J. Med..

[38] O’Neill L.A. (2006). Targeting signal transduction as a strategy to treat inflammatory diseases. Nat. Rev. Drug Discov..

[39] Yang Y., Lv J., Jiang S., Ma Z., Wang D., Hu W., Deng C., Fan C., Di S., Sun Y. (2016). The emerging role of Toll-like receptor 4 in myocardial inflammation. Cell Death Dis..

[40] Falck-Hansen M., Kassiteridi C., Monaco C. (2013). Toll-like receptors in atherosclerosis. Int. J. Mol. Sci..

