# Supplementary figures and images for: Truncated Pneumolysin from Streptococcus pneumoniae as a TLR4-Antagonizing New Drug for Chronic Inflammatory Conditions
Source: Cells. 2020 May 9;9(5):1183. doi: 10.3390/cells9051183 (PMC7290803; doi:10.3390/cells9051183)

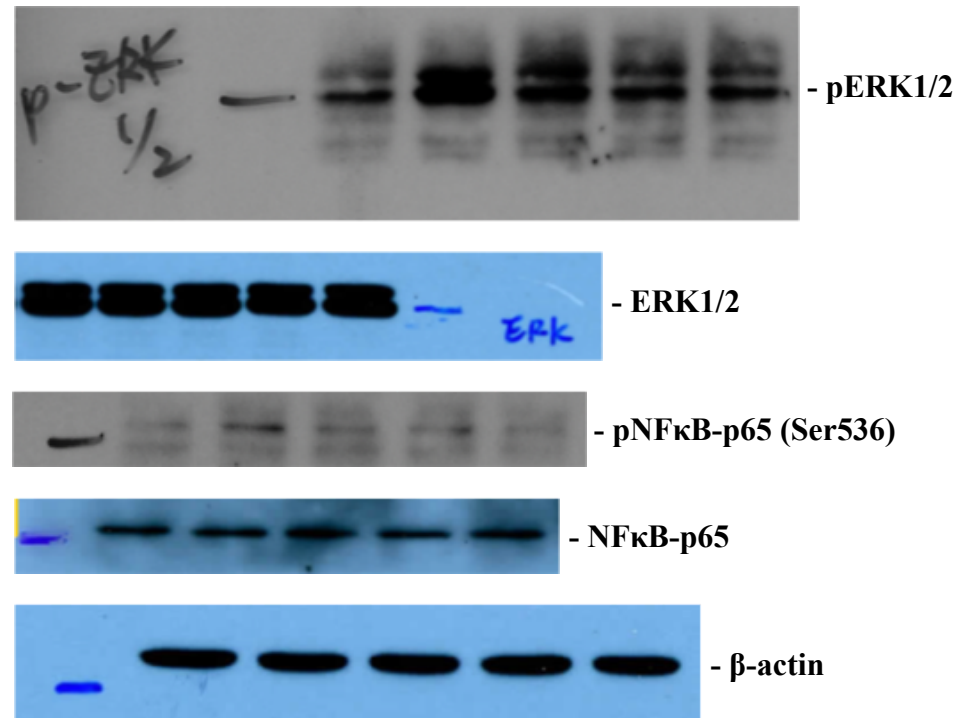

**Figure S1:** Gel images of Western blot in Figure 3C.

Supplement: Supplementary file 1 [file cells-09-01183-s001.pdf]
